# Prevalence and symptom severity of thrower’s shoulder in elite youth handball players

**DOI:** 10.1186/s13102-026-02199-2

**Published:** 2026-11-07

**Authors:** Leonard Achenbach, Anne Benning, Florian Zeman, Moritz Kaiser, Jonas Limmer

**Affiliations:** 1 Department of Orthopedics, König-Ludwig-Haus, Julius-Maximilians-University Würzburg, Brettreichstraße 11, Würzburg, 97074 Germany; 2 Center for Clinical Studies, University Medical Center Regensburg, Regensburg, Germany; 3 Department of Trauma Surgery, University Medical Center Regensburg, Regensburg, Germany

**Keywords:** Elite youth handball, Overuse shoulder symptoms, Thrower’s shoulder, Western Ontario Shoulder Instability Index (WOSI)

## Abstract

**Objective:**

To identify the prevalence and severity of overuse shoulder symptoms in elite youth handball players.

**Methods:**

Six-hundred and sixty (52.3% boys) elite youth handball players were followed prospectively for one season during the seasons 2016-17, 2017-18, 2018-19, and 2019-20. Players completed standardized questionnaires throughout the season and reported any overuse shoulder symptoms. The Western Ontario Shoulder Instability Index (WOSI) questionnaire was distributed five times per season. Latent class analysis (LCA) was carried out to identify different symptom patterns.

**Results:**

Of 660 athletes, 148 (22.4%) (50% boys, age: 14.1 ± 0.7 years) reported shoulder problems in at least one WOSI questionnaire. The average symptom severity was 457.8 ± 352.0 points (range 4 to 1834). The questions with the highest average scores were about the lack of strength (31.3 ± 27.5), the concern that the shoulder will get worse (29.5 ± 28.5), and clicking, cracking, or snapping of the shoulder (29.3 ± 30.9). No statistically significant sex-related differences were identified across the four WOSI domains.

LCA showed no discernible pattern of symptom distribution.

**Conclusion:**

The prevalence of overuse shoulder symptoms is high and presents as a multi-faceted problem in elite youth handball. The WOSI questionnaire indicates symptom severity rather than distinct symptom patterns.

## Background

Overuse injuries to the shoulder have become a growing concern in overhead sports, particularly in handball, due to the repetitive throwing motions that place high mechanical stress on the shoulder joint [6]. These injuries not only limit performance but may also jeopardize long-term athletic development and career prospects. In professional adult handball players, the prevalence of overuse-related throwing shoulder injuries has been reported to range between 26% and 36% in both sexes [12, 15]. In contrast, shoulder overuse injuries appear to be even more prevalent among elite youth handball players, with reported prevalences ranging from 25% to 60% [2, 3, 16].

Despite the high prevalence of shoulder overuse injury in elite youth handball players, little is known about the severity and specific characteristics in developing athletes, and detailed symptom-based analyses remain limited. Early identification and a better understanding of symptom presentation are essential for prevention and appropriate intervention, particularly in young athletes who are still undergoing musculoskeletal development.

To our knowledge, no previous study has systematically assessed the symptom severity specific to the throwing shoulder of elite youth handball players. Such knowledge could not only support early diagnosis but also aid the design of targeted prevention programs and rehabilitation strategies. Therefore, the aim of this prospective study was to assess the prevalence and symptom severity of shoulder complaints in elite youth handball players of both sexes. We used a validated patient-reported outcome measure to evaluate symptom patterns and severity to contribute to a more comprehensive clinical understanding of this widespread issue.

## Methods

A prospective study including 660 (345 boys and 315 girls) elite youth handball players was conducted during the 2016–2020 seasons. Participants were eligible if they were actively selected members of a federal state youth handball team at the time of the survey. Players (*n* = 1) who had undergone previous shoulder surgery or had been hospitalized due to shoulder-related complaints were excluded. The study was part of an ongoing trial exploring potential injury risk factors for overuse-related shoulder symptoms in elite youth handball.

### Baseline and overuse injury questionnaire

Data management was conducted with REDCap, a web-based clinical data management system [9]. Clinical data were collected and stored on a local server operated by the data processing center of the University of Regensburg.

The personal and anthropometric data, i.e. age, height, weight, previous shoulder injuries, previous shoulder surgery, and current shoulder symptoms of the players were collected at the beginning of the season using a standardized baseline questionnaire.

The online questionnaire assessed any throwing shoulder symptoms obtained during handball training sessions or matches. Using online survey software, a standardized questionnaire was emailed to all study participants at five predefined time points throughout the 7-month competitive season: after baseline screening, before the start of the season, after the preseason period, at mid-season, and at the end of the season. Participants who did not initially respond received an automated reminder email after one week. In addition, coaches were routinely contacted to remind the athletes to complete the questionnaires.

To assess symptoms related to shoulder function, we employed the German-translated version of the Western Ontario Shoulder Instability Index (WOSI), a validated patient-reported outcome measure specifically designed for persons with shoulder instability [7, 11]. This instrument comprises 21 items grouped into four scales: physical symptoms (10 items), sport/recreation/work (4 items), lifestyle (4 items), and emotional well-being (3 items). Each item is scored using a 100 mm visual analogue scale (VAS), resulting in a total score ranging between 0 (optimal shoulder function) and 2100 points (maximal impairment). A WOSI questionnaire was considered positive when athletes reported any degree of symptoms, defined as a score greater than zero in at least one WOSI item. The WOSI allows for a detailed assessment of both functional and psychosocial impact of shoulder problems [11]. Given its multidimensional structure, it has also been applied in broader contexts to capture shoulder problems of handball athletes [1, 15].

Informed written consent was obtained from all study participants and their parents. The study design was approved by the Ethics Committee of the University of Regensburg (ID 17-895-101).

### Statistical analysis

If more than one WOSI questionnaire had been completed by the same player, only the questionnaire with the highest score was analyzed further. Based on previous research, a threshold of 420 points (20% of the maximum score of 2100 points) was identified for further analysis [2]. Sex-related differences in the four WOSI domain scores (physical symptoms, sports/recreation/work, lifestyle, and emotional well-being) were assessed using multivariate analysis of variance (MANOVA), with sex as the between-subjects factor. Domain-specific and total WOSI scores stratified by sex are presented descriptively as mean ± standard deviation (SD).

No a priori sample size was calculated because this study included all elite youth players of the Southern German handball federations.

A latent class analysis (LCA) was conducted [5, 13, 17]. The goal of LCA is to identify and describe the hidden “typologies” or homogenous classes within a population. These classes are identified by a set of indicator variables. To identify the latent profiles of symptoms, the four scales of the WOSI (physical symptoms, sport/recreation/work, lifestyle and emotional well-being) were used as indicator variables. R version 4.3.2 with the packages depmixS4, foreign, flextable, officer, ggplot2, gridExtra, ggpubr, stats and haven were used to conduct the analysis and produce graphics. Five models ranging from one to five classes were fitted to identify the best-fitting model. The model choice was based on the following statistical criteria: AIC (with lower values indicating a better model fit), entropy, the average latent class posterior probability, and the size of the smallest class. Once the best class model had been identified, each patient was assigned to a specific class according to their posterior class membership probability.

## Results

Of 660 youth elite athletes, 148 (22.4%; 50% boys) responded positively with at least one point to at least one question on the WOSI questionnaire regarding shoulder symptoms, and were therefore included in the further analysis (Table 1).

**Table 1:** Anthropometric and handball-specific data at baseline

	Male athletes (*n* = 74)	Female athletes (*n* = 74)	Total (*n* = 148)
Age (years)	14.7 ± 0.5	13.6 ± 0.6	14.1 ± 0.7
Height (cm)	181.6 ± 7.8	169.3 ± 6.9	175.2 ± 9.6
Weight (kg)	75.4 ± 5.7	59.6 ± 8.2	67.1 ± 9.6
Handball experience (years)	8.7 ± 2.3	7.9 ± 2.1	8.3 ± 2.2

Data is given in mean ± standard deviation (SD)

The average WOSI score was 457.8 ± 352.0 points. The WOSI total score ranged from 4 to 1834 points. Of 148 athletes, 70 (47.3%) reported a minimum WOSI score of 420 points (20% of the maximum score of 2100 points) [2].

The sex-stratified domain analysis is presented in Table 2. The omnibus MANOVA showed no significant multivariate effect of sex across the four WOSI domains (Wilks’ λ = 0.987, F(4,107) = 0.355, *p* = 0.840).

**Table 2:** Domain analysis

	Male athletes (*n* = 74)	Female athletes (*n* = 74)	*p*-value
‍Physical symptoms	236.0 ± 151.4	253.7 ± 198.9	0.54
‍Sports/recreation/work	74.3 ± 66.8	87.0 ± 97.8	0.40
‍Lifestyle	47.3 ± 54.9	59.1 ± 80.7	0.35
‍Emotional well-being	79.5 ± 65.4	78.8 ± 82.1	0.70
‍Total WOSI	437.1 ± 269.1	478.6 ± 426.1	0.50

**Fig. 1:**
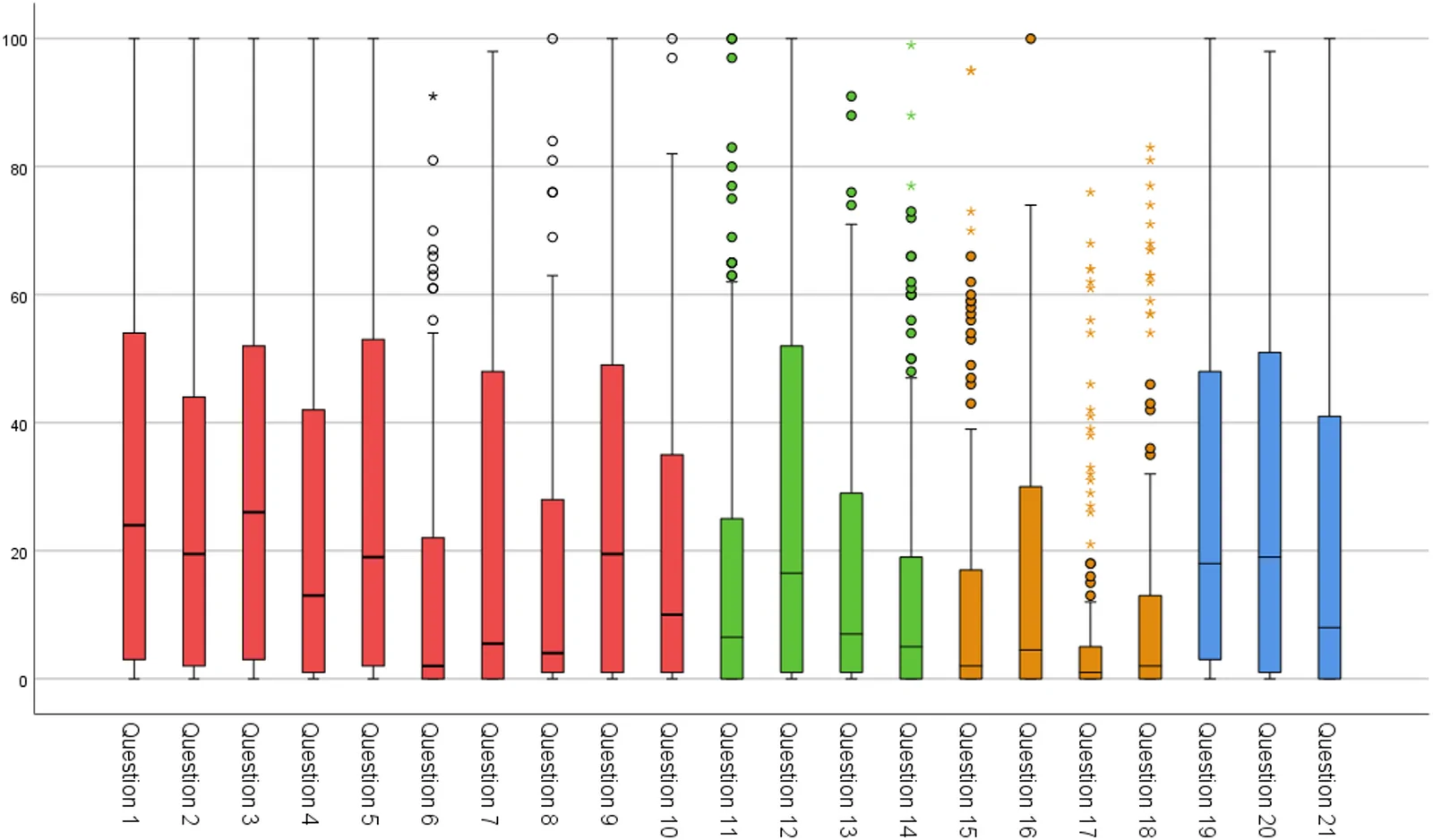
Boxplots representing the symptom severity assessed by the Western Ontario Shoulder Instability Index. The four scales are physical symptoms (red), sports, recreation, and work (green), lifestyle (orange), and emotional well-being (blue)

The three questions with the highest average scores were Question 3, 5, and 20 (Table 3; Fig. 1).

**Table 3:** Symptom severity assessed by the Western Ontario shoulder instability index

Scale	Western Ontario Shoulder Instability Index Question	Male players (*n* = 74)	Female players (*n* = 74)	Total
Physical symptoms	Question 1 “How much pain do you experience in your shoulder during overhead activities?”	25.3 ± 23.2 (0–75)	33.8 ± 30.1 (0-100)	29.1 ± 26.3 (0-100)
	Question 2 “How much aching or throbbing do you experience in your shoulder?”	25.9 ± 24.5 (0-100)	25.7 ± 25.3 (0–85)	25.5 ± 24.5 (0-100)
	Question 3 “How much weakness or lack of strength do you experience in your shoulder?”	33.2 ± 27.8 (0–87)	30.3 ± 27.8 (0-100)	31.3 ± 27.5 (0-100)
	Question 4 “How much fatigue or lack of stamina do you experience in your shoulder?”	26.9 ± 26.9 (0–78)	25.5 ± 27.9 (0-100)	25.6 ± 26.9 (0-100)
	Question 5 “How much clicking, cracking, or snapping do you experience in your shoulder?”	29.0 ± 30.4 (0-100)	31.7 ± 32.6 (0-100)	29.3 ± 30.9 (0-100)
	Question 6 “How much stiffness do you experience in your shoulder?”	14.4 ± 20.0 (0–81)	14.4 ± 24.1 (0–91)	14.7 ± 21.4 (0–91)
	Question 7 “How much discomfort do you experience in your neck muscles as a result of your shoulder problems?”	16.5 ± 21.8 (0–92)	32.0 ± 31.7 (0–98)	23.8 ± 28.6 (0–98)
	Question 8 “How much feeling of instability or looseness do you experience in your shoulder?”	17.0 ± 21.7 (0–84)	18.9 ± 25.2 (0-100)	17.1 ± 23.0 (0-100)
	Question 9 “How much do you compensate for your shoulder with other muscles?”	28.0 ± 26.4 (0-100)	28.1 ± 27.0 (0–91)	27.2 ± 26.2 (0-100)
	Question 10 “How much loss of range of motion do you have in your shoulder?”	21.9 ± 26.4 (0–97)	19.6 ± 26.1 (0-100)	21.2 ± 25.7 (0-100)
Sports, Recreation and Work	Question 11 “To what extent has your shoulder limited your participation in sports or recreational activities?”	19.2 ± 26.7 (0-100)	21.1 ± 27.5 (0-100)	19.6 ± 26.1 (0-100)
	Question 12 “How much has your shoulder affected your ability to perform the specific skills required for your sport or work? (If your shoulder affects both sports and work, consider the area that is most affected.)”	27.6 ± 28.1 (0-100)	30.1 ± 30.7 (0-100)	28.2 ± 29.0 (0-100)
	Question 13 “How much do you feel the need to protect your arm during activities?”	19.0 ± 19.5 (0–76)	20.5 ± 27.6 (0–91)	19.6 ± 26.1 (0–91)
	Question 14 “How much difficulty do you experience lifting heavy objects below shoulder level?”	14.2 ± 17.3 (0–61)	18.5 ± 26.5 (0–99)	16.0 ± 22.0 (0–99)
Lifestyle	Question 15 “How afraid are you to fall on your shoulder?”	14.3 ± 19.5 (0–70)	15.4 ± 24.0 (0–95)	13.7 ± 21.0 (0–95)
	Question 16 “How much difficulty do you experience in maintaining your desired level of fitness?”	16.7 ± 20.2 (0–70)	17.5 ± 23.5 (0-100)	16.4 ± 21.3 (0-100)
	Question 17 “How much difficulty do you have “roughhousing” or “horsing around” with family or friends?”	6.2 ± 11.4 (0–62)	11.4 ± 21.3 (0–76)	8.8 ± 17.0 (0–76)
	Question 18 “How much difficulty do you have sleeping because of your shoulder?”	11.6 ± 19.4 (0–67)	17.3 ± 25.8 (0–85)	14.1 ± 22.5 (0–85)
Emotional well-being	Question 19 “How conscious are you of your shoulder?”	27.1 ± 24.1 (0–80)	27.1 ± 28.1 (0-100)	27.5 ± 26.3 (0-100)
	Question 20 “How worried are you that your shoulder will get worse?”	31.0 ± 27.0 (0–31)	30.4 ± 31.5 (0–98)	29.5 ± 28.5 (0–98)
	Question 21 “How frustrated are you because of your shoulder problems?”	21.3 ± 25.2 (0-100)	26.6 ± 32.9 (0-100)	23.2 ± 28.5 (0-100)

Data are presented as mean ± SD (range)

**Fig. 2:**
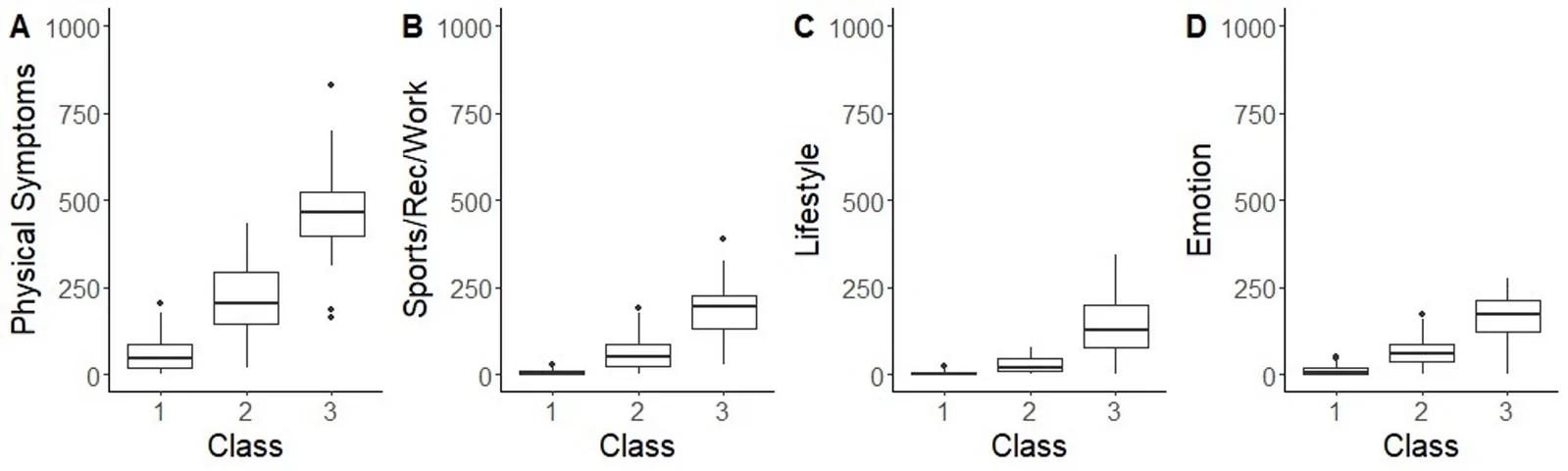
Boxplots describing the latent class analysis for the four different scales of the Western Ontario Shoulder Instability Index. **A** physical symptoms, (**B**) sports/recreation/work, (**C**) lifestyle, and (**D**) emotional well-being

**Fig. 3:**
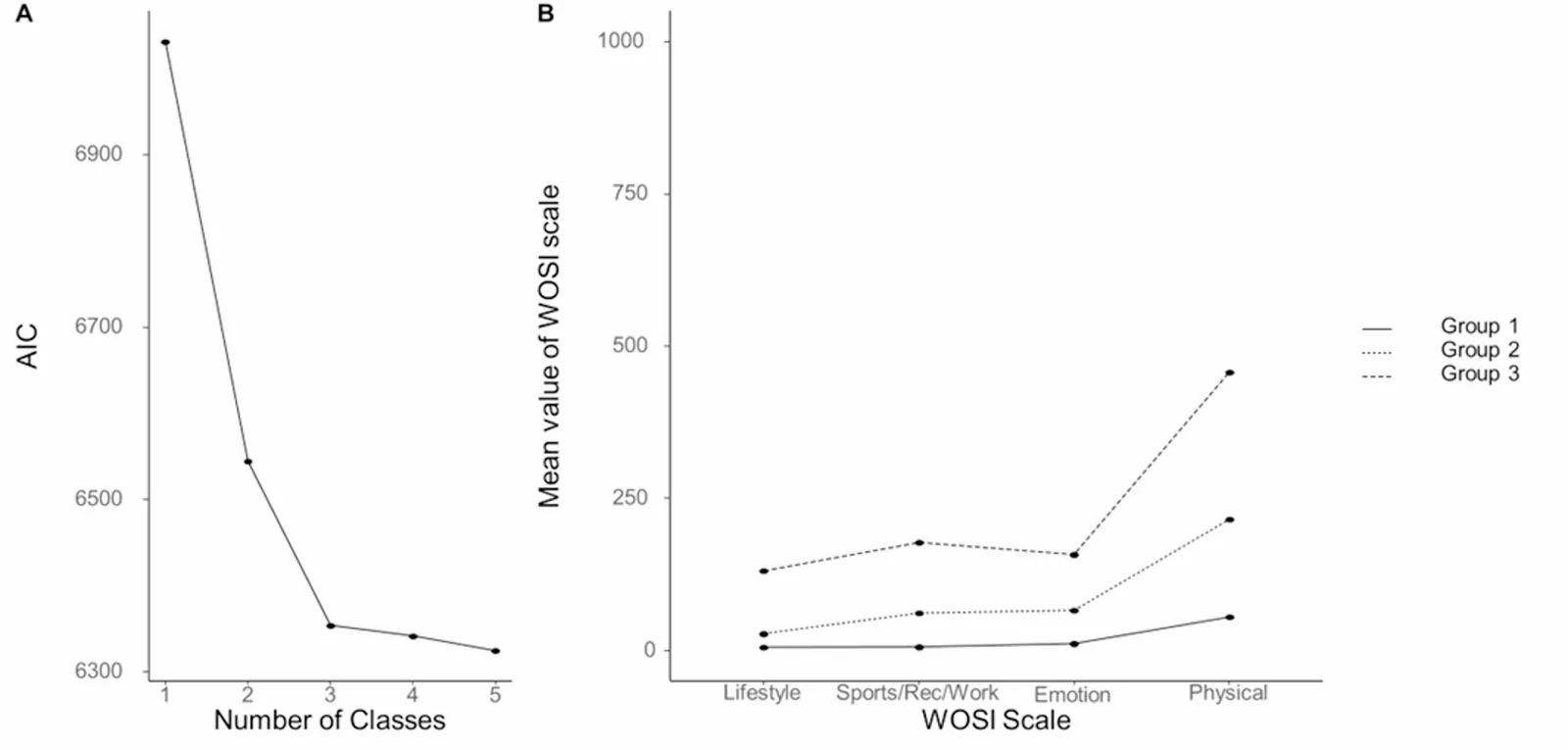
Elbow plots demonstrating the (**A**) AIC for the number of classes, and (**B**) mean value / scale relationship for the three groups

The results of the LCA did not support the existence of distinct classes of shoulder symptoms, as measured by the WOSI. Figure 2 illustrates the symptom severity profiles across the WOSI domains, while Table 4 and Fig. 3 present the model evaluation and fit criteria. 

**Table 4:** Evaluation of models

Models	LL	AIC	BIC	Smallest class count (*n*)	Smallest class size (%)	Entropy
1 Class	-3507.71	7031.42	7054.84	153	100	-
2 Class	-3255.21	6544.42	6594.19	55	36.19	0.97
3 Class	-3150.96	6353.91	6430.02	40	26.21	0.91
4 Class	-3135.89	6341.78	6444.23	28	19.31	0.70
5 Class	-3118.23	6324.46	6453.26	27	19.01	0.59

## Discussion

The most significant finding was the high prevalence of throwing-related shoulder symptoms among elite youth handball players, with 22.4% reporting a positive Western Ontario Shoulder Instability Index (WOSI) questionnaire. All four scales of the WOSI questionnaire demonstrated moderate-to-high severity scores, with an average symptom severity of 457.8 points, indicative of considerable impairment in shoulder function. Symptom severity varied across the four WOSI scales, with higher scores observed in the physical symptoms and emotional well-being scales and lower average scores in the lifestyle scale. Furthermore, 47.3% of athletes reached the 20% threshold indicative of clinically relevant functional limitation [2]. No distinct symptom patterns were identified beyond differences in overall symptom severity.

Although the diagnostic criteria indicated a three-class model, further examination of the latent classes revealed largely parallel profiles. In the context of latent class analysis, this pattern suggests that a single underlying class was effectively divided into three groups based on overall symptom severity (high, medium, and low). The LCA results therefore indicate that, apart from differences in symptom severity, no distinct subtypes with specific symptom patterns were identified. Furthermore, the findings suggest that higher symptom severity in one WOSI scale is associated with higher symptom severity across all WOSI scales.

Further research should examine whether the identified classes represent distinct and theoretically meaningful severity levels, or if they are the result of arbitrary class separation. The results suggest that subgroups identified by specific symptom patterns are unlikely to exist, regardless of symptom severity as measured by the WOSI. This finding makes the WOSI a useful tool for assessing the severity of throwing shoulder symptoms in elite youth handball players of both sexes. The distinctness of the WOSI scales should be further analyzed in future research.

At the aggregated domain level, no significant overall sex-related difference in WOSI symptom burden was identified. The multivariate analysis of the four WOSI domains was not significant, and the descriptive total WOSI score was only modestly higher in female than in male athletes. The observed difference in mean total WOSI scores between female and male athletes (41.5 points) was below previously reported standard errors of measurement (SEM) and minimal detectable change (MDC) values for the WOSI [8, 18]. Although these thresholds were established in different populations and cannot be directly applied to group comparisons, this finding provides additional context for the small descriptive difference observed in our cohort. Previous studies have nevertheless reported a higher prevalence of shoulder problems among female youth handball players [3, 4]. Moreover, both Hoppe et al. and Mashimo et al. identified playing position and sex as non-modifiable risk factors for shoulder injuries in overhead sports, with backcourt players and female players showing higher susceptibility [10, 14].

Sex may therefore remain relevant to the occurrence of shoulder problems, while the present analysis does not demonstrate a distinct sex-related pattern in symptom severity across the WOSI domains. Previous research has identified sex-specific risk factors for shoulder injuries in adolescent handball players, including reduced shoulder rotator strength in female athletes and scapular dyskinesis in male athletes [4].

Although the present study assessed self-reported symptoms rather than clinically diagnosed injuries, the observed prevalence of shoulder-related symptoms is in line with previous reports from youth handball populations, in which prevalence rates between approximately 25% and 60% have been described [2, 3, 16]. Comparisons with adult elite handball players should be interpreted with caution, as differences in physical maturity, training volume, and cumulative shoulder load may substantially influence symptom presentation and injury risk.

The study encompasses a large collective of elite youth handball players with potentially diverse overuse shoulder injuries. Using a broad questionnaire of shoulder instability, the study aimed to capture symptom severity associated with throwing motions. However, the nonspecific nature of the questionnaire may have contributed to a high correlation among injury groups, making it difficult to identify distinct (injury) patterns. Despite the extensive dataset, LCA showed no discernible pattern in the distribution of symptoms. This finding suggests that the WOSI measures a unidimensional construct that indicates symptom severity rather than distinct classes of symptom patterns. This highlights the WOSI’s usefulness in assessing the severity of functional impairment in various shoulder injuries experienced by elite handball players.

However, the lack of identifiable injury patterns underscores the complexity of overuse throwing shoulder injuries in this population. To further differentiate shoulder pain and to elucidate specific injury mechanisms, a specialized examination tailored to the unique biomechanical stresses of handball throwing motions is imperative. Assessments involving soft tissue adaptations, strength analysis, or muscle synergy analysis can provide deeper insights into the underlying pathology and may also guide targeted interventions aimed at optimizing athlete recovery and performance [2].

### Strengths and limitations

The strength of this study is the large cohort of elite youth handball players of both sexes and the comprehensive approach applied. However, several limitations should be acknowledged. First, the reliance on self-reported data, albeit collected using standardized questionnaires, introduces the potential for recall bias and subjective interpretation of symptoms. Second, the analytical approach based on the individual highest WOSI score per athlete is inherently biased toward the most severe symptom presentation. While this approach intentionally reflects peak shoulder burden, it may overestimate the average symptom severity over the season and should therefore be interpreted accordingly. Finally, the exclusion of players with a history of previous shoulder surgery or shoulder-related hospitalization represents an important limitation, as prior shoulder pathology may influence the risk of developing overuse-related shoulder symptoms. Consequently, the findings of this study primarily reflect overuse-related shoulder symptoms that are not secondary to previous shoulder surgery or hospitalization.

## Conclusion

The prevalence of overuse shoulder symptoms is high and presents as a multi-faceted problem in elite youth handball. The WOSI questionnaire is indicative of symptom severity rather than distinct symptom patterns.

## Data Availability

The datasets generated and/or analyzed during the current study are not publicly available due to participant-privacy concerns, but are available from the corresponding author on reasonable request.

## Abbreviations

AIC: Akaike-Information-Criterion
BIC: Bayesian-Information-Criterion
LCA: Latent class analysis
MDC: Minimal detectable change
SEM: Standard error of measurement
VAS: Visual analog score
WOSI: Western Ontario Shoulder Instability Index
